# Combination Oxylanthanum Carbonate and Tenapanor Lowers Urinary Phosphate Excretion in Rats

**DOI:** 10.34067/KID.0000000709

**Published:** 2025-01-22

**Authors:** Satya Medicherla, Guru Reddy, Pramod Gupta, Glenn M. Chertow, Shalabh Gupta

**Affiliations:** 1 Unicycive Therapeutics, Los Altos, California; 2 Division of Nephrology, Stanford University School of Medicine, Stanford, California

**Keywords:** CKD, ESKD, hyperphosphatemia, phosphate binders

## Abstract

**Key Points:**

- Combination therapy with oxylanthanum carbonate+tenapanor led to greater reductions in urinary phosphate excretion than either drug alone.
- We demonstrated dose-dependent and sizable reductions in urinary phosphate excretion in response to oxylanthanum carbonate monotherapy.

**Background:**

This study evaluated the combined effects of oxylanthanum carbonate (OLC), an investigational phosphate binder, and tenapanor, an approved sodium/hydrogen exchanger 3 inhibitor that reduces paracellular phosphate absorption, on urinary phosphate excretion in rats on a high-phosphorus diet.

**Methods:**

Sixty-four male Sprague Dawley rats were randomized into eight groups: vehicle; tenapanor (0.15 mg/kg) only; OLC (0.75%, 1.5%, and 3%) only; and combination OLC (0.75%, 1.5%, and 3%)+tenapanor (0.15 mg/kg). Vehicle and tenapanor were dosed orally twice per day, whereas OLC was incorporated into diets. We collected 24-hour urine samples to measure urinary phosphate excretion, a proxy for intestinal phosphate absorption efficiency. Primary analyses compared pooled results in the three OLC dose groups.

**Results:**

In the tenapanor 0.15 mg/kg group, mean urinary phosphate excretion from days 9 to 11 was 8.5 mg/d (12.5%) lower compared with the vehicle group. In the OLC-alone groups, mean urinary phosphate excretion (pooled across the 0.75%, 1.5%, and 3% OLC dose groups) was 12.1 mg/d (17.7%) lower compared with the vehicle group. Compared with vehicle, urinary phosphate excretion was 28.1 mg/d (41.3%) lower in the combination OLC+tenapanor groups (*P* = 0.016). Bliss model of independence assessing the statistical significance between observed and predicted results indicated that combination OLC+tenapanor was synergistic (*P* = 0.009 for 0.75% OLC+tenapanor and *P* = 0.010 for 1.5% OLC+tenapanor).

**Conclusions:**

We demonstrated sizable reductions in urinary phosphate excretion in response to OLC monotherapy and the most pronounced reductions in urinary phosphate excretion when using OLC in combination with tenapanor.

## Visual Abstract

**Figure:**
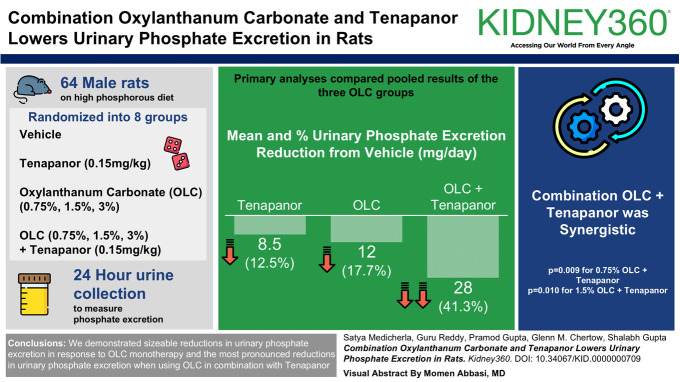

## Introduction

The kidneys are among the organs responsible for maintaining homeostasis, particularly in the realms of fluid and electrolyte, mineral metabolism, and acid–base balance. Depending on the etiology and severity of kidney disease in humans, disorders relating to lack of homeostasis vary in their manifestations. With respect to mineral metabolism, the body uses a variety of mechanisms to maintain physiological concentrations of calcium and phosphate, both of which are essential for maintaining cellular integrity and function, growth and mineralization of bone in childhood and adolescence, and the maintenance of bone health in adulthood.^1^ The population reference range for serum phosphate ranges from roughly 2.7 to 4.5 mg/dl in adults.^2^ Maintaining normal serum phosphate concentrations requires adequate dietary phosphorus intake, along with normal serum concentrations of calcitriol (1, 25-dihydroxy-vitamin D), to facilitate intestinal phosphate absorption. Persons with inadequate dietary phosphorus intake and/or vitamin D deficiency are subject to osteomalacia, often referred to as rickets in children.^3^

Hyperphosphatemia is seen in most patients requiring maintenance dialysis^4^ and in nearly all patients with ESKD who maintain adequate dietary intake because phosphorus is abundant in most protein sources. In a number of observational studies, hyperphosphatemia has been associated with all-cause mortality,^5,6^ cardiovascular events,^5,7^ and fracture.^8^ Thus, new therapeutic approaches that achieve recommended, and ideally normal, phosphorus concentrations are welcome for patients with hyperphosphatemia.

A study by King *et al.* assessed the efficacy of combination treatment with tenapanor hydrochloride, a recently US Food and Drug Administration (FDA)-approved sodium/hydrogen exchanger 3 (NHE3) inhibitor that reduces paracellular phosphate absorption by inducing conformational changes in the intestinal epithelium,^9^ and sevelamer, a phosphate binder, in rats with normal kidney function. This study found that “although both tenapanor and sevelamer reduce intestinal phosphate absorption individually, administration of tenapanor and sevelamer together results in more pronounced reductions in intestinal phosphate absorption than if either agent is administered alone.” On the basis of the promising results from King *et al.*, other combination treatments of phosphorus management should be explored.

Oxylanthanum carbonate (OLC) is an investigational new drug being developed under the FDA's 505(b)(2) regulatory pathway. If approved, OLC will share substantially the same product label and prescribing information as the reference-listed drug lanthanum carbonate. Although OLC forms the same insoluble phosphate complex (lanthanum phosphate) in the gastrointestinal tract as lanthanum carbonate, OLC tablets are smaller in size, swallowed whole with water, and not chewed. The objective of the study was to evaluate the effects of combination OLC+tenapanor on urinary phosphate excretion in rats on a high-phosphorus diet. If dietary phosphorus is bound and/or its absorption is otherwise blocked, less phosphate will be available for absorption, and if less phosphate is absorbed in the intestinal tract, less is available for urinary excretion. As such, urinary phosphate excretion is the best experimental proxy for the efficiency of intestinal absorption of dietary phosphorus.

## Methods

### Animals

Animal experiments were conducted in a fully Association for Assessment and Accreditation of Laboratory Animal Care-accredited animal facility at Gubra, Inc., Denmark, and in accordance with Gubra's bioethical guidelines, which are fully compliant with internationally accepted principles for the care and use of laboratory animals. All experimental protocols for animal studies were approved by the Institutional Animal Care and Use Committee (2023-15-0201-01393). Sixty-four male Sprague Dawley rats of 9 weeks old (11 weeks by the end of the study) were fed standard chow 1 week before study start. On study day 1, all animals were switched to high-phosphorus diets consisting of chow spiked with 0.4% inorganic phosphorus ([1:1 sodium:potassium salt—wt./wt.] 1.1% total phosphorus content) for the rest of the study.

### Study Design

**Figure 1:**
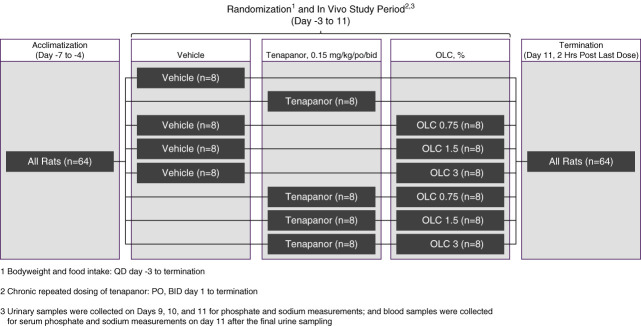
**Study consisted of acclimatization, randomization, an *in vivo* study period, and termination.** Animals were acclimated from day −7 to day −4. Body weight and food intake were measured daily from study day −3 until termination. On study day −1, animals were randomized into eight groups: one vehicle; one tenapanor (0.15 mg/kg); three OLC (0.75%, 1.5%, and 3%); and three combination treatment with OLC and tenapanor (OLC 0.75%, 1.5%, and 3%+tenapanor 0.15 mg/kg). On study day 1, animals were switched to high-phosphorus diets. During the *in vivo* study period (days 1–11), vehicle and tenapanor were dosed orally twice per day, whereas OLC was incorporated into the diets. Urine samples were collected on days 9, 10, and 11 for phosphate measurements. Animals were euthanized on day 11 two hours after the last dose. BID, twice a day; OLC, oxylanthanum carbonate; PO, per oral gavage; QD, once a day.

The study consisted of acclimatization, randomization, an *in vivo* study period, and termination (Figure 1). Animals were acclimated from day −7 to day −4. On study day −1, animals were randomized into eight study groups on the basis of body weight: one vehicle; one tenapanor (0.15 mg/kg) only; three OLC (0.75%, 1.5%, and 3%) only; and three combination treatment with OLC and tenapanor (OLC 0.75%, 1.5%, and 3%+tenapanor 0.15 mg/kg). During the *in vivo* study period (days 1–11), vehicle and tenapanor were dosed orally (po) twice daily, whereas OLC was incorporated into diets. The study was terminated after animals were euthanized on day 11 two hours after the last dose.

### Study Assessments

Body weight and food intake were measured daily from study day −3 until termination. Twenty-four–hour urine samples were collected using metabolic cages on days 9, 10, and 11 for phosphate and sodium measurements. Urine was centrifuged at 2000×*g* for 2 minutes, aliquoted into sample tubes, and stored at −70°C. On study day 11 after the final urine sampling, blood samples were collected for measurement of serum phosphate and sodium. Urine and serum samples were measured using a commercial kit (Roche Diagnostics) on the Cobas C501 autoanalyzer.

### Statistical Analyses

Mean urinary phosphate excretion for each animal from days 9 to 11 was averaged by treatment group. While we examined effects separately in each OLC dose group, our primary analyses compared pooled results in the three OLC dose groups combined. We compared pooled results using one-way ANOVA, followed by the Games–Howell test, and individual groups with vehicle using one-way ANOVA, followed by Dunnett's multiple comparison test. A threshold of *P* < 0.05 was used to determine significant differences between groups. We used Statistical Package for the Social Sciences for all statistical analyses.

We used the Bliss model of independence to determine whether the combination of OLC and tenapanor was independent, synergistic, or antagonistic.^10^ The Bliss model compares the predicted combination response, calculated using the complete additivity of probability theory on the basis of the observed effect of each individual agent administered alone, with the observed effect of combination treatment.^10^ We calculated the mean percentage reduction in urinary phosphate excretion relative to vehicle by treatment group by averaging urinary phosphate excretion for each animal from days 9 to 11. We conducted paired *t* tests to assess the statistical significance between observed and predicted results. We considered two-tailed *P* values < 0.05 to be statistically significant.

## Results

### Effect on Body Weight and Food Intake

**Figure 2:**
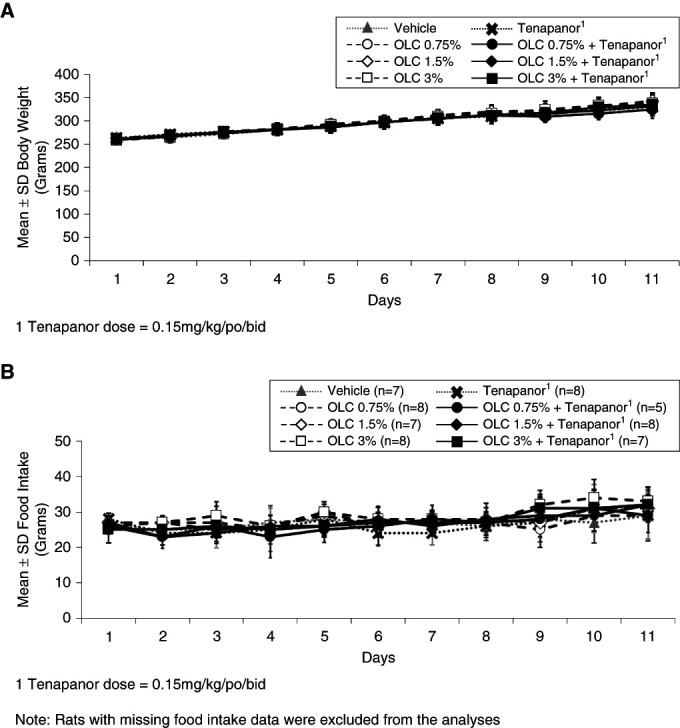
**Body weight and food intake were not significantly affected by OLC, tenapanor, or combination treatment compared with the vehicle group.** (A) Mean±SD body weight by treatment groups—day 1–11 (*n*=8 per treatment group). (B) Mean±SD food intake by treatment groups—day 1–11.

Body weight (Figure 2A) and food intake (Figure 2B) were not significantly affected by OLC, tenapanor, or combination treatment compared with vehicle.

### Effect on Urinary Phosphate Excretion

**Figure 3:**
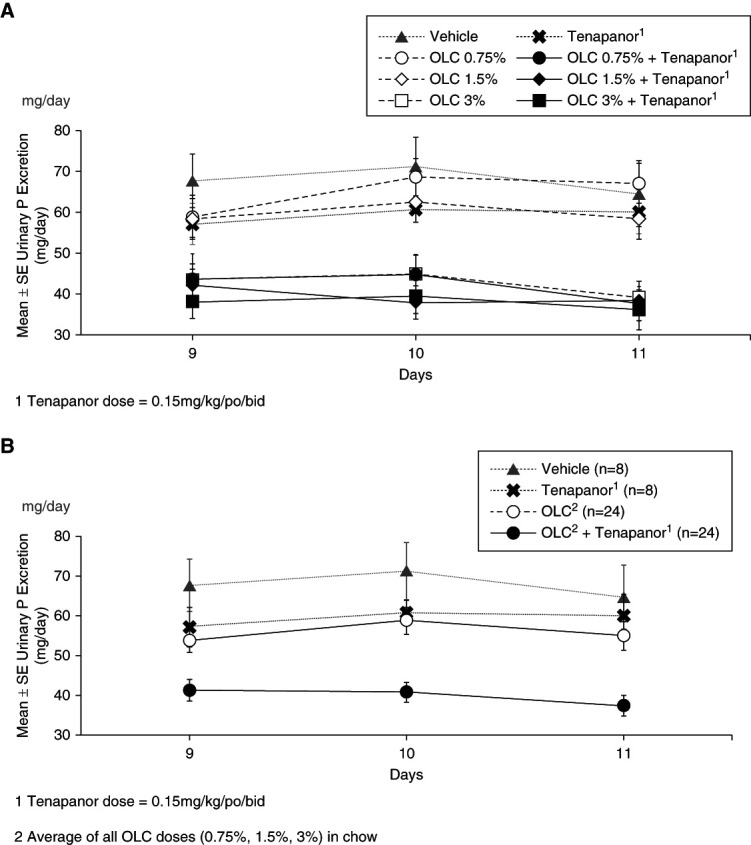
**OLC dose dependently decreased urinary phosphate excretion.** When used together, OLC+tenapanor decreased urinary phosphate excretion such that combination reductions were larger compared with vehicle, OLC only, and tenapanor only. (A) Mean±SE urinary phosphate excretion—days 9, 10, and 11 (*n*=8 per treatment group). (B) Mean±SE urinary phosphate excretion—days 9, 10, and 11. SE, standard error; Urinary P, urinary phosphate.

Twenty-four–hour urinary phosphate excretion for each dose group over the final 3 days of treatment is shown in Figure 3. OLC decreased urinary phosphate excretion in a dose-dependent manner. When used together, OLC+tenapanor decreased urinary phosphate excretion such that combination reductions were larger compared with vehicle, OLC only, and tenapanor only.

**Figure 4:**
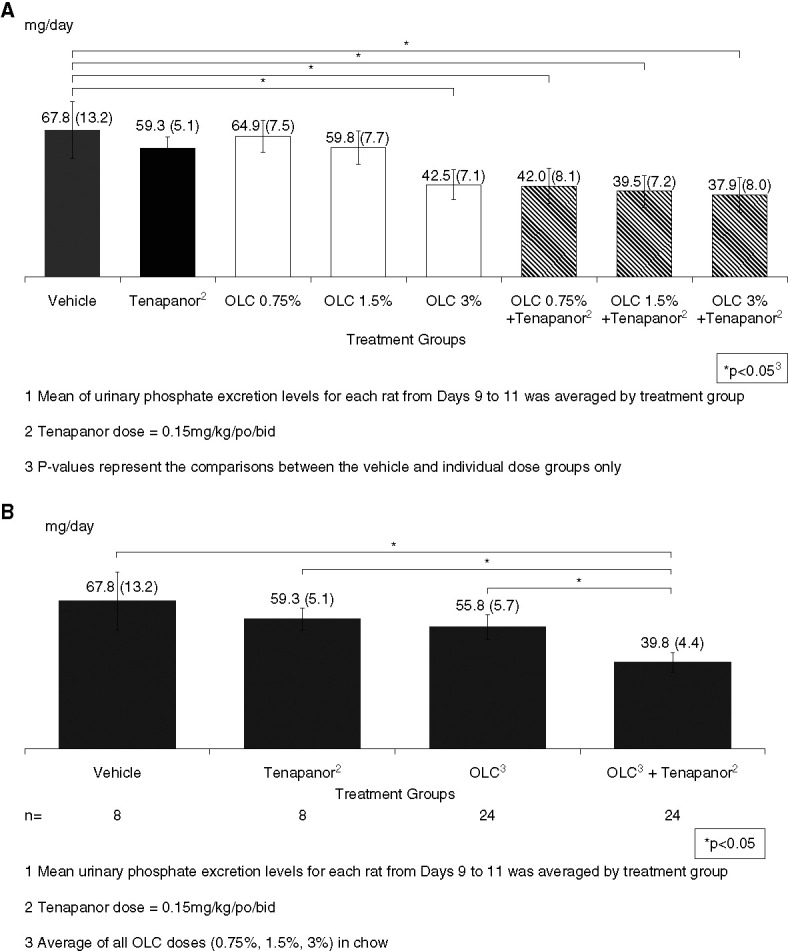
**In the tenapanor 0.15 mg/kg group, mean urinary phosphate excretion from days 9 to 11 was 8.5 mg/d lower compared with the vehicle group.** In the OLC-alone groups, mean urinary phosphate excretion (pooled across the 0.75%, 1.5%, and 3% OLC dose groups) was 12 mg/d lower compared with the vehicle group. In the OLC+tenapanor groups (pooled across the 0.75%, 1.5%, and 3% OLC dose groups), urinary phosphate excretion was 28 mg/d lower compared with the vehicle group. OLC+tenapanor significantly decreased urinary phosphate excretion compared with OLC only and tenapanor only. (A) Mean^1^ (±95% CI) urinary phosphate excretion by treatment groups—days 9–11 (*n*=8 per treatment group). (B) Mean^1^ (±95% CI) urinary phosphate excretion by treatment group—days 9 to 11. Statistical comparisons were conducted using one-way ANOVA with Games-Howell Test. CI, confidence interval.

Mean urinary phosphate excretion from day 9 to 11 by each dose group is shown in Figure 4A, with results pooled across the three OLC doses shown in Figure 4B. In the tenapanor 0.15 mg/kg group, mean urinary phosphate excretion from days 9 to 11 was 8.5 mg/d (12.5%) lower compared with the vehicle group. In the OLC-alone groups, mean urinary phosphate excretion (pooled across the 0.75%, 1.5%, and 3% OLC dose groups) was 12.1 mg/d (17.7%) lower compared with the vehicle group. In the OLC+tenapanor groups (pooled across the 0.75%, 1.5%, and 3% OLC dose groups), urinary phosphate excretion was 28.1 mg/d (41.3%) lower compared with the vehicle group (*P* = 0.016) (Supplemental Table 1).

**Figure 5:**
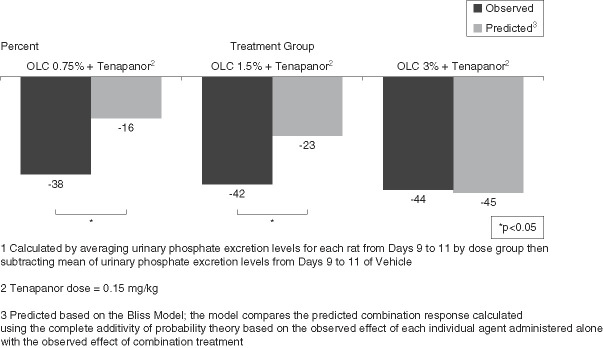
**Observed reduction in urinary phosphate excretion for the OLC+tenapanor groups was larger than the predicted reduction on the basis of the single-agent effects in accordance with the Bliss model.** The difference in observed (38%) versus predicted (16%) urinary phosphate excretion was most pronounced for the combination group with the lowest OLC dose (0.75%).

The observed reduction in urinary phosphate excretion for the OLC+tenapanor groups (0.75% and 1.5% OLC doses) was significantly larger than the predicted reduction on the basis of the single-agent effects in accordance with the Bliss model (*P* = 0.009 for 0.75% OLC+tenapanor and *P* = 0.010 for 1.5% OLC+tenapanor; Figure 5). The difference in observed (38%) versus predicted (16%) urinary phosphate excretion was the most pronounced for the combination group with the lowest OLC dose (0.75%). The observed versus predicted reductions in urinary phosphate excretion for the OLC 1.5% and 3% groups were 42 versus 23% and 44 versus 45%, respectively.

### Effect on Urinary Sodium Excretion

Combination OLC+tenapanor significantly reduced urinary sodium excretion on day 11 only.

### Effect on Serum Phosphate Levels

No significant differences in serum phosphate concentrations were observed between the groups on day 11.

## Discussion

The ultimate goal of phosphate management therapies is to achieve and maintain recommended, and ideally normal, serum phosphate concentrations in patients with CKD/ESKD. FDA-approved phosphate management therapies include phosphate binders and tenapanor, an NHE3 blocker that diminishes transcellular phosphate absorption. Calcium-based phosphate binders (typically calcium acetate or carbonate) are commonly prescribed and can help correct hypocalcemia that often accompanies CKD–mineral and bone disorder. However, clinical practice guidelines advise against more than modest use of calcium-based phosphate binders because they themselves can contribute to dystrophic calcification^11^ and, in the presence of relatively low bone turnover (as is commonly seen among older persons), can result in hypercalcemia, particularly when delivered in conjunction with calcitriol^12^ or active vitamin D analogs, such as doxercalciferol or paricalcitol.^13^ Lanthanum carbonate is an effective phosphate binder that reduces serum phosphate by binding dietary phosphorus in the gastrointestinal tract. Lanthanum has the highest relative phosphate-binding coefficient (2.0) compared with other binders (sevelamer hydrochloride: 0.75, calcium acetate: 1.0, anhydrous magnesium carbonate: 1.7, hydrated magnesium carbonate: 1.3, aluminum hydroxide: 1.5, and aluminum carbonate: 1.9).^14^ A lack of low bone turnover has also been observed in patients on dialysis treated with lanthanum carbonate, in contrast to those treated with calcium carbonate,^15^ indicating that lanthanum may improve bone health.

Despite adherence with the rigors of peritoneal and hemodialysis sessions, typically daily and thrice weekly, respectively, and the use of one or more phosphate binders at doses of two to three, or often four or more, tablets or capsules three times daily with meals, fewer than half of patients reach the Kidney Disease Improving Global Outcomes–recommended target serum phosphate concentration^11^ in any given month, with even far fewer reaching that goal on a consistent basis.^16^ Many studies have found an association between elevated serum phosphate concentrations and increased morbidity (*e.g*., accelerated vascular and heart valve calcification^17^ and dystrophic calcification^18^) and mortality.^19^ In particular, seminal experimental work by Giachelli *et al.* and others demonstrated that in the presence of physiological or higher concentrations of phosphate, vascular smooth muscle cell dedifferentiate, demonstrating phenotypic characteristics of osteoblasts.^20^ Clinical studies demonstrate accelerated vascular and heart valve calcification in patients with ESKD,^17^ and patients with higher serum phosphate concentrations demonstrate the most severe degrees of dystrophic calcification.^18^ Thus, there is an urgent unmet need for new phosphate management strategies.

The combination of OLC and tenapanor may support enhanced inhibition of intestinal phosphate absorption by leveraging two distinct mechanisms of action. Our study used a nearly identical study design and dosing regimen as a previous study conducted by King *et al.*,^21^ which found that when administered together, sevelamer and tenapanor decreased urinary phosphate excretion significantly more than either sevelamer or tenapanor alone across all sevelamer dose levels.

In this study, we demonstrated potent effects of the novel lanthanum-based phosphate binder OLC and synergistic effects when given in conjunction with tenapanor, an NHE3 blocker that diminishes transcellular phosphate absorption. OLC, with or without tenapanor, significantly reduced urinary phosphate excretion in rats fed a diet high in phosphorus, and combination OLC+tenapanor synergistically reduced urinary phosphate excretion in rats compared with either treatment alone. With respect to demonstrated synergy, sample sizes within groups were small, but it is noteworthy that the most pronounced synergy (the greatest difference between predicted and observed urinary phosphate excretion) was evident in the lowest OLC dose group (0.75%). One potential explanation is that at higher OLC doses, there may be relatively little phosphate remaining in the intestinal lumen, and as a result, there may be limited incremental benefit of tenapanor. It should also be noted that the OLC+tenapanor combination exhibited four- to seven-fold more synergistic effects compared with the sevelamer+tenapanor combination.^21^ However, the comparison of synergistic effects for combination OLC+tenapanor versus combination tenapanor+sevelamer is based on a dosing efficacy calculation that may not reflect the complexities of phosphate management. Because the experiments were not contemporaneous, direct comparisons on the magnitude of urinary phosphate excretion reduction between studies should not be entertained. The potency of OLC and the dosing regimen of tenapanor (one small tablet twice daily) could significantly reduce pill burden, below the typical nine to eighteen phosphate binder capsules or tablets ingested by patients with moderate-to-severe hyperphosphatemia in current practice. On the basis of the promising results of this study, we would expect OLC to reduce intestinal phosphate absorption in humans, promoting loss of phosphate in stool and abrogating or ameliorating complications associated with hyperphosphatemia. Further study in patients with CKD or ESKD and hyperphosphatemia is warranted.

Our study had several limitations. First, we sought to assess the effects of combination OLC+tenapanor on rats with normal kidney function rather than kidney failure. We acknowledge that mechanisms regulating phosphate balance differ between healthy persons and those with CKD/ESKD. However, use of healthy rats may remove potential confounding factors stemming from physiological changes due to kidney disease or kidney failure, and previous studies have used healthy rats to assess the efficacy of phosphate binders and tenapanor.^22–24^ In addition, our study used urinary phosphate excretion to measure phosphate-binding capacity. Although urinary phosphate excretion is not a direct measure of intestinal phosphate absorption, previous studies have demonstrated that decreases in urinary phosphate excretion are associated with increases in fecal phosphate, which is a direct measure of intestinal absorption, thus providing additional support for urinary phosphate excretion as an appropriate and accepted marker of intestinal phosphate absorption in healthy rats.^23,25^ Another limitation of this study is that rats were fed a high-phosphorus diet and patients with CKD typically follow a low-phosphorus diet. In patients with CKD whose dietary phosphorus is not typically elevated as in the rat model, low intestinal phosphate levels after the introduction of one phosphate-binding agent (*e.g*., OLC) may limit the potential for additive effects or synergy of the combination treatment. Finally, the simultaneous initiation of a high-phosphorus diet and combination OLC+tenapanor may have prevented the development of hyperphosphatemia, limiting the ability to assess potential dose response in the setting of hyperphosphatemia. Future studies should address these limitations by assessing the effects of combination OLC+tenapanor on serum phosphate concentrations in rats with CKD and in a population of patients with CKD and hyperphosphatemia.

In summary, we used three doses of OLC to compare OLC alone, tenapanor alone, and the combination of OLC+tenapanor in rats fed a high-phosphorus diet. We demonstrated dose-dependent and sizable reductions in urinary phosphate excretion (a proxy for intestinal phosphate absorption) in response to OLC monotherapy and the most pronounced reductions in urinary phosphate excretion when using OLC in combination with tenapanor. Studies in patients with ESKD and hyperphosphatemia will be required to understand the most effective and best tolerated OLC-containing regimens as we aim to improve long-term control of hyperphosphatemia and prevent its myriad associated complications.

## Supplementary Material

SUPPLEMENTARY MATERIAL

## Data Availability

All data are included in the manuscript and/or supporting information; Partial restrictions to the data and/or materials apply. Data are generated in Gubra, Hørsholm Kongevej 11B, DK-2970 Hørsholm, Denmark. Aggregated summary data underlying this article are available upon reasonable request to the corresponding author. Interested parties must sign a data use agreement to access data.

## Supplemental Material

Supplemental Table 1. *Post hoc* Games–Howell test results comparing mean urinary phosphate excretion^1^ in vehicle, OLC^2^, tenapanor^3^, and OLC^2^+tenapanor^3^.
