# Supplementary material for: Combination Oxylanthanum Carbonate and Tenapanor Lowers Urinary Phosphate Excretion in Rats
Source: Kidney360. 2025 Jan 22;6(3):361–8. doi: 10.34067/KID.0000000709 (PMC11970866; doi:10.34067/KID.0000000709)
Supplement: SUPPLEMENTARY MATERIAL [file kidney360-6-361-s001.pdf]

ASN American Society of Nephrology | JASN Journal of the American Society of Nephrology | KIDNEY360 Accessing Our World From Every Angle | CJASN Clinical Journal of the American Society of Nephrology

ASN Journal Disclosure Form

As per ASN journal policy, I have disclosed any financial relationships or commitments I have held in the past 36 months as included below. I have listed my Current Employer below to indicate there is a relationship requiring disclosure. If no relationship exists, my Current Employer is not listed.

G. Chertow reports the following:
Employer: Stanford University School of Medicine; Consultancy: Akebia, Ardelyx, AstraZeneca, Beren, CalciMedica, Calico, Miromatrix, Panoramic, Sanifit, Toku, Unicycive, Vertex; Ownership Interest: Ardelyx, CloudCath, Durect, DxNow, Eliaz Therapeutics, Outset, Renibus, Unicycive; Research Funding: NIDDK, NIAID, CSL Behring; Advisory or Leadership Role: Board of Directors, Satellite Healthcare, Co-Editor, Brenner & Rector's The Kidney (Elsevier); and Other Interests or Relationships: DSMB service: NIDDK, Aethlon, Bayer, Mineralys, ReCor.

I understand that the information above will be published within the journal article, if accepted, and that failure to comply and/or to accurately and completely report the potential financial conflicts of interest could lead to the following: 1) Prior to publication, article rejection, or 2) Post-publication, sanctions ranging from, but not limited to, issuing a correction, reporting the inaccurate information to the authors' institution, banning authors from submitting work to ASN journals for varying lengths of time, and/or retraction of the published work.

Name: Glenn M. Chertow
Manuscript ID: K360-2024-000655R1
Manuscript Title: Combination Oxylanthanum Carbonate and Tenapanor Lowers Urinary Phosphate Excretion in Rats
Date of Completion: November 27, 2024
Disclosure Updated Date: June 18, 2024

ASN American Society of Nephrology | JASN Journal of the American Society of Nephrology | KIDNEY360 Accessing Our World From Every Angle | CJASN Clinical Journal of the American Society of Nephrology

ASN Journal Disclosure Form

As per ASN journal policy, I have disclosed any financial relationships or commitments I have held in the past 36 months as included below. I have listed my Current Employer below to indicate there is a relationship requiring disclosure. If no relationship exists, my Current Employer is not listed.

P. Gupta reports the following:
Employer: Unicycive Therapeutics; Ownership Interest: GE; ABBV; ABT; BAX; UNCY; Patents or Royalties: UNCY; and Advisory or Leadership Role: UNCY.

I understand that the information above will be published within the journal article, if accepted, and that failure to comply and/or to accurately and completely report the potential financial conflicts of interest could lead to the following: 1) Prior to publication, article rejection, or 2) Post-publication, sanctions ranging from, but not limited to, issuing a correction, reporting the inaccurate information to the authors' institution, banning authors from submitting work to ASN journals for varying lengths of time, and/or retraction of the published work.

Name: Pramod Gupta
Manuscript ID: K360-2024-000655R1
Manuscript Title: Combination Oxylanthanum Carbonate and Tenapanor Lowers Urinary Phosphate Excretion in Rats
Date of Completion: December 5, 2024
Disclosure Updated Date: May 20, 2024

ASN
American Society of Nephrology
JASN
Journal of the American Society of Nephrology
KIDNEY360
Accessing Our World From Every Angle
CJASN
Clinical Journal of the American Society of Nephrology

ASN Journal Disclosure Form

As per ASN journal policy, I have disclosed any financial relationships or commitments I have held in the past 36 months as included below. I have listed my Current Employer below to indicate there is a relationship requiring disclosure. If no relationship exists, my Current Employer is not listed.

S. Gupta reports the following:
Employer: Unicycive Therapeutics, Inc.; Ownership Interest: Unicycive Therapeutics, Inc.; Research Funding: Unicycive Therapeutics, Inc.; Patents or Royalties: Unicycive Therapeutics, Inc.; and Advisory or Leadership Role: Unicycive Therapeutics, Inc.

I understand that the information above will be published within the journal article, if accepted, and that failure to comply and/or to accurately and completely report the potential financial conflicts of interest could lead to the following: 1) Prior to publication, article rejection, or 2) Post-publication, sanctions ranging from, but not limited to, issuing a correction, reporting the inaccurate information to the authors' institution, banning authors from submitting work to ASN journals for varying lengths of time, and/or retraction of the published work.

Name: Shalabh Gupta
Manuscript ID: K360-2024-000655R1
Manuscript Title: Combination Oxylanthanum Carbonate and Tenapanor Lowers Urinary PhosphateExcretion in Rats,
Date of Completion: November 28, 2024
Disclosure Updated Date: May 20, 2024

ASN
American Society of Nephrology
JASN
Journal of the American Society of Nephrology
KIDNEY360
Accessing Our World From Every Angle
CJASN
Clinical Journal of the American Society of Nephrology

ASN Journal Disclosure Form

As per ASN journal policy, I have disclosed any financial relationships or commitments I have held in the past 36 months as included below. I have listed my Current Employer below to indicate there is a relationship requiring disclosure. If no relationship exists, my Current Employer is not listed.

S. Medicherla reports the following:
Employer: Unicycive Therapeutics Inc.; Ownership Interest: Unicycive Therapeutics Inc; AAPL, NVDA, AMXN, TSLA and others; and Advisory or Leadership Role: Siripharma Labs, Inc.

I understand that the information above will be published within the journal article, if accepted, and that failure to comply and/or to accurately and completely report the potential financial conflicts of interest could lead to the following: 1) Prior to publication, article rejection, or 2) Post-publication, sanctions ranging from, but not limited to, issuing a correction, reporting the inaccurate information to the authors' institution, banning authors from submitting work to ASN journals for varying lengths of time, and/or retraction of the published work.

Name: Satya Medicherla
Manuscript ID: K360-2024-000655
Manuscript Title: Combination Oxylanthanum Carbonate and Tenapanor Lowers Urinary Phosphate Excretion in Rats"
Date of Completion: November 26, 2024
Disclosure Updated Date: May 20, 2024

ASN
American Society of Nephrology
JASN
Journal of the American Society of Nephrology
KIDNEY360
Accessing Our World From Every Angle
CJASN
Clinical Journal of the American Society of Nephrology

ASN Journal Disclosure Form

As per ASN journal policy, I have disclosed any financial relationships or commitments I have held in the past 36 months as included below. I have listed my Current Employer below to indicate there is a relationship requiring disclosure. If no relationship exists, my Current Employer is not listed.

G. Reddy reports the following:
Employer: Unicycive Therapeutics ; Genentech (Spouse employer);; and Ownership Interest: Unicycive Therapeutics; Genentech.

I understand that the information above will be published within the journal article, if accepted, and that failure to comply and/or to accurately and completely report the potential financial conflicts of interest could lead to the following: 1) Prior to publication, article rejection, or 2) Post-publication, sanctions ranging from, but not limited to, issuing a correction, reporting the inaccurate information to the authors' institution, banning authors from submitting work to ASN journals for varying lengths of time, and/or retraction of the published work.

Name: Guru Reddy
Manuscript ID: K360-2024-000655R1
Manuscript Title: Combination Oxylanthanum Carbonate and Tenapanor Lowers Urinary PhosphateExcretion in Rats
Date of Completion: November 25, 2024
Disclosure Updated Date: November 25, 2024
