# Supplementary material for: Combination Oxylanthanum Carbonate and Tenapanor Lowers Urinary Phosphate Excretion in Rats
Source: Kidney360. 2025 Jan 22;6(3):361–8. doi: 10.34067/KID.0000000709 (PMC11970866; doi:10.34067/KID.0000000709)
Supplement: SUPPLEMENTARY MATERIAL [file kidney360-6-361-s002.pdf]

Supplementary Table: Post-hoc Games-Howell Test Results Comparing Mean Urinary Phosphate Excretion[1] in Vehicle, OLC[2], Tenapanor[3], and OLC[2]+Tenapanor[3]

Treatment Group (A)	Treatment Group (B)	Mean Difference (A-B)	Std. Error	Sig.	95% Confidence Interval	
					Lower Bound	Upper Bound
Vehicle	Tenapanor[3]	8.510	7.215	0.653	-13.997	31.017
	OLC[2]	12.093	7.327	0.397	-10.441	34.626
	OLC[2] + Tenapanor[3]	28.053	7.090	0.016	5.698	50.409
Tenapanor[3]	Vehicle	-8.510	7.215	0.653	-31.017	13.997
	OLC[2]	3.583	3.884	0.793	-7.134	14.299
	OLC[2] + Tenapanor[3]	19.543	3.417	<0.001	9.892	29.195
OLC[2]	Vehicle	-12.093	7.327	0.397	-34.626	10.441
	Tenapanor[3]	-3.583	3.884	0.793	-14.299	7.134
	OLC[2] + Tenapanor[3]	15.961	3.647	<0.001	6.216	25.706
OLC[2] + Tenapanor[3]	Vehicle	-28.053	7.090	0.016	-50.409	-5.698
	Tenapanor[3]	-19.543	3.417	<0.001	-29.195	-9.892
	OLC[2]	-15.961	3.647	<0.001	-25.706	-6.216

1 Mean Urinary phosphate excretion levels for each rat from Days 9 to 11 were averaged by treatment group
2 Average of all OLC doses (0.75%, 1.5%, 3%) in chow
3 Tenapanor dose = 0.15 mg/kg/po/bid
